# Outbreak of Poliomyelitis — Somalia and Kenya, May 2013

**Published:** 2013-06-14

**Authors:** World Health Organization, Div of Global Migration and Quarantine, National Center for Emerging and Zoonotic Infectious Diseases, Div of Viral Diseases, National Center for Immunization and Respiratory Diseases, Global Immunization Div, Center for Global Health, CDC

On May 9, 2013, the Somalia Ministry of Health and the World Health Organization (WHO) reported a confirmed wild poliovirus type 1 (WPV1) case in a girl aged 32 months from Mogadishu (Banadir Region), with onset of acute flaccid paralysis (AFP) on April 18, 2013. Subsequently, eight additional WPV1 cases have been confirmed in Somalia, seven in Banadir Region and one in Bay Region. These are the first reported polio cases in Somalia since March 2007.

On May 16, 2013, the Kenya Ministry of Public Health and Sanitation and WHO reported a confirmed WPV1 case with onset on April 30, 2013, in a girl aged 4 months from the Dadaab refugee camps near the Somalia border. Four additional cases were confirmed in the camps. These are the first reported polio cases in Kenya since July 2011. All data are as of June 11, 2013.

Genetic sequence analysis of isolates from both countries indicates the isolates are closely related, with evidence of a single introduction of virus into the region and subsequent local transmission before detection. These viruses are both closely related to WPV1 currently circulating in West Africa.

In Somalia, a rapid response polio supplementary immunization activity (SIA) was conducted May 14–17 in all 16 districts of Banadir Region. A subsequent SIA was conducted May 26–29 in a larger geographic area of Somalia, and SIAs are planned for June, July, and August. In Kenya, the first SIA in the Dadaab refugee camps and the surrounding three districts was conducted May 27–30. Subsequent SIAs with increasing geographic coverage in Kenya are planned for June, July, and August. Preventive SIAs are being conducted in areas of Ethiopia and Yemen, and surveillance for AFP is being strengthened in all countries in the Horn of Africa.

Poliovirus is spread person-to-person through fecal-oral contact and through contaminated water. For every WPV1 case with paralysis, approximately 200 asymptomatic infected susceptible persons are also shedding poliovirus (1). In 2012, only 223 polio cases were reported globally, the fewest ever reported in a calendar year (2). As of June 11, a total of 50 polio cases had been reported in 2013 globally, compared with 67 cases reported during the same period in 2012 (3).

CDC recommends that all international travelers complete polio vaccination before travel. For travelers to countries with designated polio risk, including Ethiopia, Kenya, and Somalia, CDC recommends an additional polio vaccine booster dose (4). CDC has issued guidelines requiring that all refugees from Kenya scheduled for U.S. resettlement receive 3 doses of oral polio vaccine regardless of age before departure for the United States, with a 2-week hold after the third dose. CDC also recommends that all refugees from Kenya who have arrived since the beginning of April 2013 receive 1 inactivated poliovirus vaccine dose regardless of vaccination history.
