# Erratum

**Published:** 2013-06-21

## Vol. 62, No. 23

In the report, “Notes from the Field: Outbreak of Poliomyelitis — Somalia and Kenya, May 2013,” the last sentence should have read as follows: “CDC also recommends that all refugees **aged <18 years** who have arrived from Kenya since the beginning of April 2013 receive 1 inactivated poliovirus vaccine dose regardless of vaccination history.”

